# Azoximer bromide and hydroxyapatite: promising immune adjuvants in cancer

**DOI:** 10.20892/j.issn.2095-3941.2023.0222

**Published:** 2024-02-05

**Authors:** Jean-François Rossi, Patrick Frayssinet, Maksim Matciyak, Nikolai Tupitsyn

**Affiliations:** 1 Institut du Cancer Avignon-Provence, Sainte Catherine – Department of Hematology-Biotherapy, Avignon 84918, France; 2 University of Montpellier, UFR Médecine, Montpellier 34090, France; 3 HASTIM Inc., Rue de Caulet, Toulouse 31300, France; 4 Petrovax NPO Pharm, Moscow 123112, Russia; 5 Laboratory of Immunology of Hematopoiesis, N.N. Blokhin Cancer Research Center (RCRC), Moscow 123112, Russia

**Keywords:** Immune adjuvants, cancer, azoximer bromide, hydroxyapatite, Toll receptor agonists, immunotherapy

## Abstract

Immune adjuvants are immune modulators that have been developed in the context of infectious vaccinations. There is currently a growing interest in immune adjuvants due to the development of immunotherapy against cancers. Immune adjuvant mechanisms of action are focused on the initiation and amplification of the inflammatory response leading to the innate immune response, followed by the adaptive immune response. The main activity lies in the support of antigen presentation and the maturation and functions of dendritic cells. Most immune adjuvants are associated with a vaccine or incorporated into the new generation of mRNA vaccines. Few immune adjuvants are used as drugs. Hydroxyapatite (HA) ceramics and azoximer bromide (AZB) are overlooked molecules that were used in early clinical trials, which demonstrated clinical efficacy and excellent tolerance profiles. HA combined in an autologous vaccine was previously developed in the veterinary field for use in canine spontaneous lymphomas. AZB, an original immune modulator derived from a class of heterochain aliphatic polyamines that is licensed in Russia, the Commonwealth of Independent States, and Slovakia for infectious and inflammatory diseases, is and now being developed for use in cancer with promising results. These two immune adjuvants can be combined in various immunotherapy strategies.

## Introduction

According to the definition of the National Cancer Institute (NCI) in the United States, an immune adjuvant is a “drug that stimulates the immune system to respond to disease”^1^. Usually, an immunologic adjuvant is a “substance used in the context of a vaccine to help boost the immune response to a vaccine so that less vaccine is needed”^2^. Thus, immune adjuvants have been developed to aid the humoral response to infectious vaccines, representing one of the most significant advances in vaccine research before the recently developed mRNA vaccines against COVID-19^3^. It has been reported that the best biological activity of these compounds is to elicit both humoral and cellular responses by initiating the cross-presentation pathway^4^. In the context of cancer, such substances are reappearing with the development of vaccine therapy in oncology^5^. We consider immune adjuvants as all substances added to any cancer therapy that amplifies an immune response against a tumor, including delivery systems and immune stimulants. However, these substances must present a very good tolerance profile due to use in a combined strategy. In this way, the clinical development of drugs is more difficult due to the search for a synergistic effect. Thus, the biological activity must mimic an amplified, but controlled inflammatory/immune reaction, and detoxification activity that can positively interfere with the tumor microenvironment (TME). Use of immune adjuvants could also be necessary in cases of immune deficiency modulated by conventional anti-cancer drugs. Few molecules, such as azoximer bromide (AZB) and hydroxyapatite (HA) ceramics, have been developed in this field and have the above properties. In this review we have attempted to better define these new immune modulators with respect to efficacy/tolerance balance, thus opening new perspectives in combined therapies against cancer.

## Immune adjuvants were first developed for anti-infectious vaccines

Immune adjuvants have been developed for vaccination against infectious diseases.

Immune adjuvants are defined as substances that increase the immunogenicity of a vaccine formulation when added to or mixed with the vaccine. The choice of an adjuvant is based on various criteria, including immunologic targeting and ability to stimulate strong humoral and cellular immunity essential for protection against certain pathogens^6^. Additionally, the balance between adjuvant properties and adverse effects has a critical role in selection. The immune adjuvant can be classified according to physicochemical properties, origin, and mechanisms of action, including delivery systems and immune modulators. Delivery systems generate a local inflammatory response associated with innate cell recruitment, while being considered antigen carriers^7^. Among the delivery systems, emulsion adjuvants, such as lipid particles, activate nuclear factor-kappa (NFκ) B and promote the production of chemokines and cytokines^3,7,8^. Emulsion adjuvants are only used in combination with vaccines and not as medicines. Immune modulators activate the immune response *via* pattern-recognition receptors (PPRs) or directly *via* cytokine secretion. Immune modulators include synthetic double-strand RNA (dsRNA), poly(I:C), virosomes, lipopolysaccharide (LPS), flagellin, imidazoquinolines, and saponins. Poly(I:C) triggers Toll-like receptor (TLR) 3, flagellin, and TLR5. Other LPS derivatives are TLR4 agonists and imidazoquinolines, such as imiquimod, which are TLR7/8 agonists^7–9^. The recent development of mRNA vaccines has changed the use of traditional vaccines. Additionally, advances in nanotechnology have involved messenger (m)RNA vaccine delivery vehicles to support higher efficacy, as observed in prospective studies that include non-diseased populations^10,11^. The mRNA molecules serve as an immunogen and adjuvant due to the intrinsic immunostimulatory properties of such a vaccine. Indeed, the intramuscular injection of the vaccine is followed by transfection of muscle cells, epidermal cells, and tissue resident immune cells, such as macrophages, dendritic cells (DCs), Langerhans cells, and immune cells, which leads to potential activation of the innate and adaptative immune systems^7^. Thus, the concept of immune adjuvants has evolved towards more precise biological activity, particularly with respect to new types of vaccines.

## Biological activities of immune adjuvants

### Dynamics of the inflammatory/immune response

**Figure 1:**
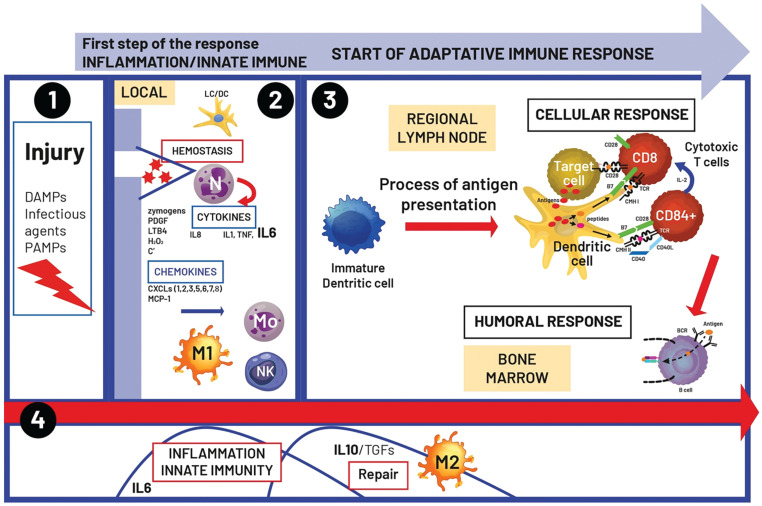
Dynamics of the inflammatory/immune response. 1. After infection or injury, different signals, such as damage-associated molecular patterns (DAMPs) or pathogen-associated molecular patterns (PAMPs), are detected by different receptors, including pathogen recognition receptors (PRRs). 2. This initiation step leads to the activation of numerous cells involved in the start of the inflammatory response, such as neutrophils (N), monocytes (Mo), macrophages (M1), natural killer cells (NK), and Langherhans cell-like dendritic cells (LC/DC), with production of chemokines [chemokine C-X-C motif ligand (CXCL)], interleukins (IL), tumor necrosis factor (TNF), and monocyte chemoattractant protein-1 (MCP-1). 3. After the initiation step, the appropriate immune response takes place at the level of the draining lymph node with the process of antigen presentation and the differentiation of dendritic cells (DC) allowing the specific immune response (cellular and humoral). 4. The initial inflammatory response is rapidly balanced by immune suppression and repair and mainly mediated by transforming growth factor (TGF)-β and IL-10.

Can we consider all the molecules that activate or restore an immune response and support the activity of immunotherapy to be immune adjuvants? If we follow the concept of immune adjuvants based on the history of anti-infectious vaccination, the notion of adjuvants is linked to an additional activity that must not be toxic, reduce boosters, and stimulate the innate immune system bridging the adaptive response to amplify and prolong the immune response, thus making antigens more effective^6^. As shown in **Figure 1**, immune adjuvants should be considered more active immune modulators to link the innate and adaptive immune responses that are particularly effective in the initiation period but with regulated activity, which are transient, allow a return to baseline, and limit toxicity^12^.

### Immune adjuvants in the dynamics of the immune response

The immune response, whatever the target, is a cellular response associated with specific or targeted cytotoxicity and a humoral response that amplifies the cytotoxicity^13^. Most of the molecules labeled as immune adjuvants have an activity located at the level of the initiation of this response by mimicking the usual signals of the stimulation of an anti-infectious response. These adjuvants are known to stimulate the innate immune effectors very rapidly at the injection site, followed by the migration of activated cells to the regional lymph nodes where the adaptive immune response is amplified. Stimulation of pathogen-associated molecular patterns (PAMPs) and danger signal pathways, including TLR and caspase-1, and lysosomal destabilization-Syk/Card9 lead to the recruitment of T and B lymphocytes and antigen presenting cells (APCs), such as DCs^6^. The nature of the innate immune signal directs the type of antigen-specific T-lymphocyte phenotypes. Secretion of interleukin (IL)-18 by macrophages promotes the development of IFN-gamma-secreting CD4 lymphocytes^14^. In contrast, IL-6 or IL-12 secreted by APCs promotes the expansion of a T-lymphocyte of the follicular-helper phenotype, supporting the secretion of IgG by B-lymphocytes^6,15–17^. Adjuvants that incorporate TLR ligands have the ability to stimulate the expansion of T-cell clones with better T-cell receptor (TCR) affinity. These TLR-based adjuvants require an additional adapter protein, myeloid differentiation primary response (MyD)88, which stimulates APCs and B cells for antibody production^17^. Moreover, MyD88 signaling also induces the formation of a germinal center that is essential for the production of antibody-secreting cells. Finally, the central activity of an immune adjuvant is to target the formation, maturation, or amplification of DCs to activate cytotoxic T lymphocyte activity. The role of cytokines, such as granulocyte-macrophage colony-stimulating factor (GM-CSF), has been reported to promote the development and maturation of myeloid cells, differentiation and survival of DCs *in vitro*, T-cell activation, and humoral responses^18^. In combination with different drugs, such as rituximab, GM-CSF has been shown to improve responses in different cancers, including follicular lymphoma^19^. GM-CSF has been associated with different immune therapies, including vaccination processes, like sipuleucel-T (Provenge^®^; Dendreon Inc., Seal Beach, CA, USA) in prostate cancer^20^. In the face of such activity, GM-CSF could be considered as an immune adjuvant.

Thus, the biological targets of an immune adjuvant are mainly the initial phases of the immune response, amplifying the first steps of the innate immune response *via* the antigen presentation process and participating in the restoration of normal immune activity in the context of immune deficiency.

## Bio-clinical context for using immune adjuvants in cancer

### Combat cancer-related immune deficiency

The growth of cancer cells is associated with a failure of immune surveillance, which represents the specific and primary immune deficiency observed in cancer, by two main strategies: avoiding immune recognition; and generating an immunosuppressive TME. Cancer cells may lose the expression of different molecules for antigen-presenting process and NK surface activators. The generation of an immunosuppressive TME implicates the secretion of suppressive molecules, the expression of inhibitory checkpoint molecules, and the induction of the recruitment of immunosuppressive cells^21,22^. Conventional cancer treatments, such as chemotherapy and radiotherapy, induce host-mediated local and systemic responses that may facilitate or support cancer progression^23^. Constitutive immune deficiency and immune exhaustion due to advanced age may also be an additional cause of immune evasion or immune resistance^24^. In addition to natural killer (NK) cells and T-lymphocyte abnormalities observed in the elderly, APCs are also reduced and functionally impaired^25^. In a mouse model using aged DCs generated from bone marrow, nucleotide-binding oligomerization domain (NOD)2 and stimulator of interferon genes protein (STING) agonists, only had a moderate effect on DC activation, while nanoparticles and micelles had no effect alone^24^. However, when nanoparticles and micelles were combined with a TLR9 agonist, a reduction in pro-inflammatory cytokine production was observed, while maintaining increased production of T cell activation of cytokines and enhancing cell surface marker expression^24^. Persistent dysregulated inflammation, as observed in patients with high levels of C-reactive protein (CRP)/interleukin-6 (IL-6) lead to immune exhaustion and tolerance, thereby blocking the cytotoxicity of active killer cells^13^. One example of this functional immune deficiency was observed involving the humoral response in patients with a B-cell malignancy and hypogammaglobulinemia or receiving rituximab after vaccination, including post-mRNA vaccination^26^.

A careful analysis of the causes of immunodeficiency in cancer patients, and especially the part associated with the disease and the patient, is of great interest.

The need of immunomodulators or immunoadjuvants administered systematically might be necessary for these patients as part of more specific immunoadjuvant therapy or in specific windows of opportunity, such as the use of anti-infectious vaccination or new opportunities for cancer vaccines.

### Modulate the TME

**Figure 2:**
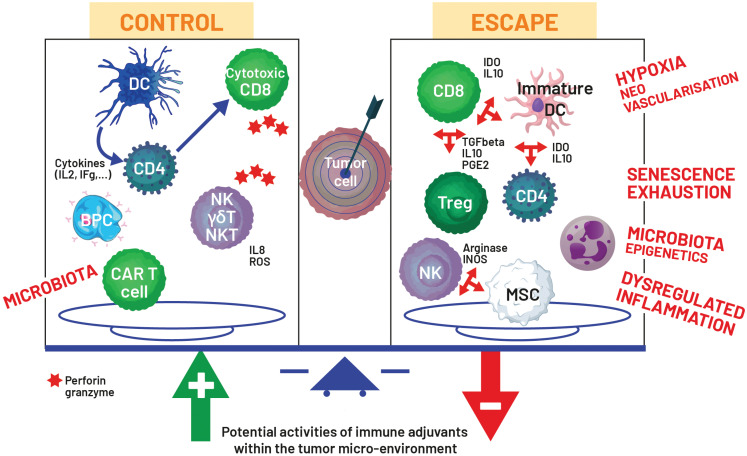
Tumor microenvironment and potential activities of immune adjuvants. The tumor microenvironment combines immune cells, which facilitate escape from immune surveillance, and cells that can eliminate cancer cells due to cytotoxic activities. Generally, the balance goes in the direction of tumor progression and the role of an immune adjuvant helps reverse this negative balance. Immune effector cells (IECs) to control cancer cells include dendritic cells (DC), CD4, cytotoxic CD8, natural killer (NK), NKT, γδ T-cells, B- and plasma cells (BPC), and chimeric antigen receptor (CAR) T-cell used therapeutically. IECs that promote cancer cell growth include subpopulations of CD4, CD8, NK, and myeloid suppressive cells (MSC). Several factors influence the tumor microenvironment, including the state of the immune system, cytokines, and chemokines, which are secreted by IEC, such as interleukin (IL) or interferon (IF) gamma (g). The microbiota can be present in the microenvironment as in gastrointestinal cancers or indirectly influences the IECs.

As shown in **Figure 2**, the TME is composed of immune effector cells (IECs) and activity factors in balance with cells that control cancer growth and others that promote tumor growth. Moreover, it has been recently identified that the microbiota influences cell initiation and development of cancer cells and modulates the immune TME through direct presence around the cancer cells and release of various factors from the tissue-dependent microbiota, especially in the intestinal tract^27,28^.

The goal of cancer immunotherapy is to block tumor immune evasion and restore immune surveillance against cancer. There are different options to achieve such a goal, including a sufficient number of IECs, recognition affinity with tumor antigens or tumor-associated antigens, homing towards cancer cells, and the reduced immunosuppression usually associated with an unfavorable TME^13^. In the field of cancer immunotherapy, cancer cytotoxicity is achieved by cytotoxic cells and activated and/or amplified by a set of recognition and communication systems that must be forced in this context. Immunoediting in cancer integrates three different stages starting by elimination with a favorable ratio between pre-malignant/malignant cells and innate cells in the context of an inflammatory response, then equilibrium and finally escape with persistent dysregulated inflammation leading to upregulation of inhibitory immune checkpoints, and production of tolerant cells and immunosuppressive cytokines giving rise to a complex TME^13^. Thus, biological targeting must consider the dynamics of the cancer, including the ratio between tumor burden and the status of the immune system. Given the biological activity of the majority of immunoadjuvants, it is possible that immunoadjuvants might be more active in pre-malignant diseases to help in the initial period of cancer immunoediting, to control minimal residual disease, to improve efficacy of cancer vaccines, or to stimulate the immune system particularly in the context of secondary immunodeficiency. Complementary activities promote a limitation of tolerance positively influencing the microenvironment, such as antioxidant activity.

Among the many possibilities offered to modulate the TME, such as drugs to reverse dysmetabolic processes, and to limit immune suppressive molecules, such as TGF-β and modulators of neovascularization, immune adjuvants represent an alternative or additional therapeutic pathway.

### Amplify the activity of anti-cancer vaccines

Immune adjuvants in the treatment of cancer have a complementary and synergistic role to the usual immunotherapy treatments. It is therefore necessary to demonstrate that immune adjuvants support biological and clinical efficacy resulting in an improvement in the response rate with a prolonged duration of response and associated with a very good tolerance profile. Like preventative anti-infectious vaccines, therapeutic cancer vaccines use patient and specific tumor antigen adjuvants to enhance the immune response. Tumor antigens include tumor-associated antigens, such as oncofetal antigens, oncoviral antigens, and neoantigens, which are usually derived from somatic mutations that characterize abnormal gene expression products, holding the high hope of inducing T-cell tumor-specific responses^14,29,30^. Tumor antigens can be made into tumor vaccines through a variety of candidate vaccine platforms, including DNA, RNA, peptides, DCs, and viral vectors^31^. One of the first cancer vaccines authorized by the U.S. Food & Drug Administration (FDA) was sipuleucel-T, which consists of autologous peripheral blood mononuclear cells (PBMCs) obtained from the patient and activated *ex vivo* using a recombinant fusion protein, PA2024, a prostate antigen that is fused to GM-CSF, active as specifically activated APCs^20^. The clinical efficacy was limited to a 4-month difference in overall survival compared to docetaxel with a 33% reduction in the risk of death and associated with T lymphocyte stimulation^32^. This therapy was the first DC immunotherapy approved by the FDA (2010) and the European Medicines Agency (EMA) (2015), although it was withdrawn from commercialization due to a request from the marketing authorization holder (Dendreon UK Ltd.), which notified the European Commission of its decision to permanently discontinue the marketing of the product for commercial reasons. The successful development of mRNA vaccines in COVID-19 has led to the rapid transfer of methodology to the cancer setting. With this technology, as the identification of the onco-antigens is made, the production is simple and rapid on a large scale leading to immunogenicity, especially since the mRNA itself has immunogenic properties and can also function as an immune adjuvant^31^. The identification and choice of a neoantigen to be incorporated into an anti-cancer vaccine strategy obey several criteria, including the quality of the identification on a frozen tissue section, the link with the capture and restriction of major histocompatibility complex (MHC) molecules, the clonality of the mutation, the presentation of the epitopes, and the stability of the interaction between the mutant peptide and the MHC molecules; all of these criteria were included in an algorithmic prediction^33^. Thus, the majority of epitope prediction focuses on the MHC I binding epitope, with most of the binding epitopes sequences containing 8–11 amino acids. However, CD4 lymphocytes play a major role in controlling tumor growth and MHC II epitope binding is included in some algorithmic prediction. Various systems have been developed to optimize transfection and mRNA stability, including addition of the 5′cap structure as a protective structure, modification of the 5′- and 3′-UTRs which are located on the flanks of the coding region, poly(A) tail modification, a major regulator of gene expression and codon modification in the open reading frame (ORF) sequence^33^. As observed with mRNA vaccines used in infectious vaccines, cancer vaccines are activated the immune system *via* the same pathways, including pattern recognition receptors, and activation of DCs to initiate signals to produce pro-inflammatory factors representing an adjuvant-like role. This excessive innate immune sensing of mRNA *via* the production of large amounts of interferons, such as type I IFNs, can lead to translation stagnation, degradation of RNA, and severe systemic side effects, such as autoimmunity. Various methods have been proposed to modulate the immunogenicity of transcription mRNA *in vitro*, including purification, modification of mRNA sequences, and addition of adjuvants. Among the methods, the TLR agonists that activate TLR3, TLR4, TLR5, TLR7, TLR9, the lipid A analog [monophosphoryl lipid A (MPLA)] as a TLR4 agonist, and imiquimod as a TLR7 agonist, have been approved for clinical use by the FDA. STING agonists represent another family and can currently be delivered by nanoparticles. As mentioned earlier, GM-CSF has been reported to promote local recruitment and activation of DCs, leading to promotion of tumor antigen presentation^18^. Some mRNA carriers, such as cationic lipids and protamine, have been reported to stimulate pro-inflammatory cytokines or TLR7/8^33^. Following the description of DC maturation, we used *ex vivo* loading of DCs by tumor cell lysates in follicular lymphoma with clinical responses in 3 of 11 patients but with technical challenges due to the need to obtain a sufficient amount of tumor proteins from a tumor sample^13,34^. However, new technology with mRNA vaccines may support a new application for such cellular therapy. Lipid nanoparticles, which are mainly composed of ionizable amino lipids, polyethylene glycol, phospholipids, and cholesterol, are most often used in cancer vaccines, as was done for COVID-19. Peptide-based deliveries, virus-like replicon particles, and cationic emulsions are also used. All these adjuvants are usually associated with the vaccine, such as certain polymer-based carriers. Few molecules are administered as medicines independent of the vaccine. This category of drugs has the advantage of modulating the effectiveness of the immune adjuvant and conveying additional effects. Among the effects, the antioxidant effect and modulation of immune tolerance leading to disruption of the immune barrier supported by the tumor micro-environment, have a major impact on the efficacy of a multi-targeting approach, especially for solid tumors. Chinese herbal medicines have been reported to exhibit anticancer activity through enhancement of the immune response, including regulation of the innate immune system, which includes macrophages, myeloid-derived suppressor cells (MDSCs), and NK cells, and the adaptive immune system, which includes CD4+ and CD8+ T cells^35^. Seventy-three clinical trials were referred to the NCI clinical.gov site^36^. Various immune adjuvants are being tested, most of which are derived from natural components, to reverse the suppressive TME to an anti-TME or to enhance the cancer vaccine-mediated immune response^29^. QS-21 is one of the active fractions from the bark of the Chilean tree, *Quillaja saponaria*, that is mainly used in different vaccine therapies for various cancers, including prostate cancer, breast cancer, and small cell lung carcinoma^37^. Montanide, a family of molecules developed for veterinary vaccines, was associated with NY-ESO OLP4. Other molecules include alun (alhydrogel or aluminium hydroxide gel) and GPI-0100, which have also been associated with different vaccines in advanced cancers. Some of the molecules were obtained from traditional Chinese medicines, such as *Rhizoma bolbostemmatis*, which were developed as immune adjuvants that modify the cancer microenvironment to heighten the anti-cancer response^29,35,37,38^.

The new development of cancer vaccines and their recent promising success paves the way for the additional use of immune adjuvants depending on the mechanism of action and the immune status of the patient^39,40^.

## Immune adjuvants used as cancer drugs: the HA and AZB examples

### HA

#### Biochemistry and biological activity

Mineral immune adjuvants, generally considered delayed adjuvants, included aluminum hydroxide or phosphate and calcium phosphate^41^. The French Institute Pasteur obtained vaccines with high immune efficiency by absorbing highly purified anatoxins on calcium phosphate; the choice of the specific calcium phosphate was crucial^41^. The different HA [Ca_10_(PO_4_)6(OH_2_)] ceramics are biocompatible and widely used in human surgery as bone substitutes or as thin layers on the surface of metals to improve bone integration and fully degradable by cells of the monocyte cell lineage^42^. Mineral adjuvants, like HA, are known to signal NOD-like receptor family, pyrin domain containing 3 (NLRP3) that activates caspase converting IL-1β and IL-18 precursors into active molecules, a similar mechanism that occurs in acute intra-articular chondrocalcinosis^43,44^. Some of these HA ceramics have been shown to have the ability to activate monocytic cells associated with high levels of secreted cytokines and chemokines, but at a lower level than occurs with LPS^45^. Similarly, for LPS, these HA powders also have the ability to enhance DC maturation by increasing HLA-DR expression. HA powders have been shown to locally attract monocytes and macrophages following intradermal or subcutaneous injection^44^. Unlike alun, HA ceramics are not associated with the immunoglobulin (Ig)E response, are biodegradable, and can fix proteins by ion exchange generally involving an anionic phosphate group with formation of Ca^2+^ complexes with the proteins. HA is used in many biotechnology processes to purify proteins from biologic solutions by ion exchange chromatography (personal data). HAs associate various proteins, including heat shock proteins (HSP), which bind the small peptides they chaperone (HSPPC). HSPs have been shown to possess some immune adjuvant effect by promoting the maturation of APCs, especially DCs, in part through TOLR enhancing antigen-presenting ability and NK stimulation^44^. Among them, HSP70, HSP90, and HSP96, have been used in HSP complexes in a cancer vaccine^45,46^ and are present in HA protein complexes^47^.

#### Clinical activity in cancer

**Figure 3:**
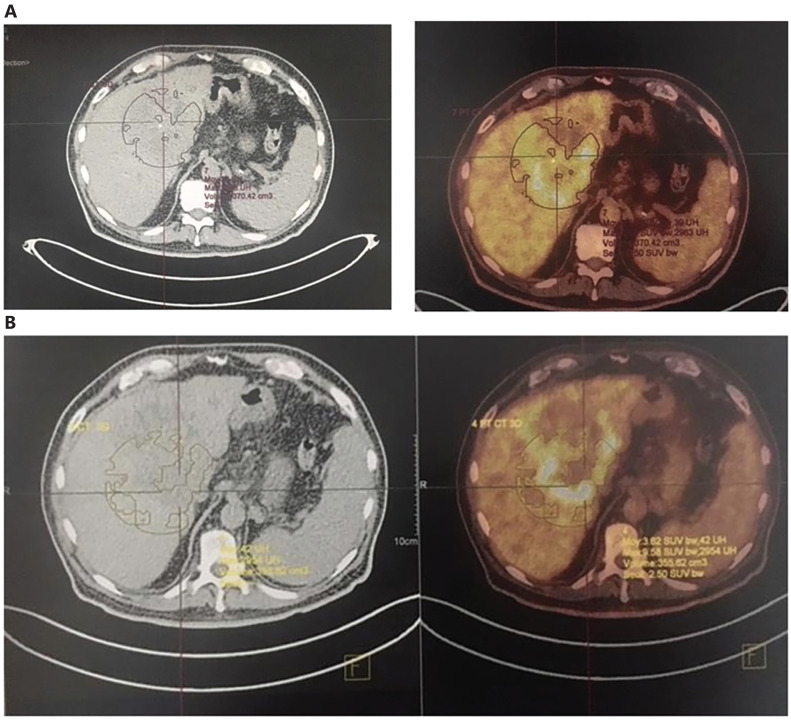
An 84-year-old man with cholangiosarcoma treated with only autologous serum vaccine (APAVAC^®^), A: CT-scan and FDG pet-scan before treatment and B: evaluation after 4 doses of sub cutaneous injection of APAVAC^®^ once per week.

**Figure 4:**
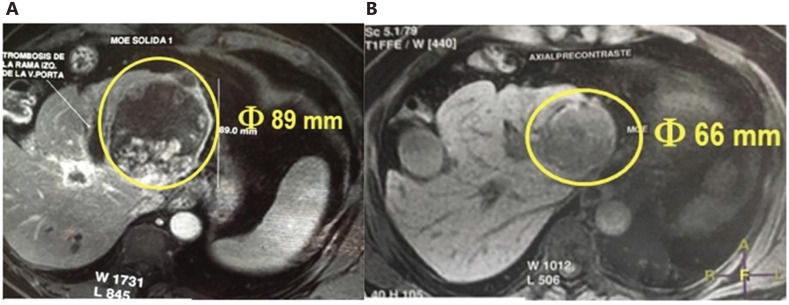
A 70-year-old patient with hepatocarcinoma treated by APAVAC^®^, A: before treatment and B: after 3 months of therapy, once per week for 4 injections followed by once monthly with a partial response.

Because of these properties, an autologous vaccine therapeutic approach has been developed in the model of spontaneous diffuse large B-cell lymphomas in dogs^48^. A prospective randomized, placebo-controlled study, double-blind study of HSPPCs–HA plus chemotherapy *vs.* chemotherapy alone was conducted that demonstrated a significant difference in time to progression (TTP, 304 days *vs.* 41days, *P = 0.0004*)^48^. This first study was extended to a large cohort of dogs with lymphoma, confirming the earlier data^49^. In humans, the HA-vaccine has been administered to patients with various cancers in a compassionate study, showing good tolerability both locally and systematically, and clinical responses were observed, including stable disease in 25% of the patients with advanced metastatic cancers (renal carcinoma, breast carcinoma, and astrocytoma) and a partial response in 15% of the patients (breast carcinoma and astrocytoma). The most encouraging results were reported in patients with recurrent disease; 4 patients (20%) were disease free after administration of the vaccine^45^. Additional observations included responses in patients with recurrent bladder cancer, and elderly patients with advanced cholangiocarcinoma receiving APAVAC^®^ alone, as shown in **Figure 3A** (before treatment) **and 3B** (after one injection per week for 1 month), and in **Figure 4A** (before treatment) **and 4B** (after 3 months of therapy, once per week for 4 injections followed by once monthly) with partial responses and necrosis.

Thus, HA represents a novel class of immune adjuvants that opens new possibilities in combined approaches for cancer vaccine treatment, as developed by Hastim Inc. (https://www.hastim.fr, Toulouse, France).

### AZB

#### Biochemistry and biological activity

AZB [Polyoxydonium^®^; (https://petrovax.com) NPO PetrovaxPharm, Moscow, Russia] is a high-molecular weight active (60–100 kDa) ternary co-polymer of 1,4-ethylene piperazine, 1,4-ethylene piperazine-N-oxide, and (N-carboxymethylene)-1,4-ethylene piperazinium bromide. AZB is synthesized by partial oxidation of the parent polymer with hydrogen peroxide to introduce N-oxide groups, followed by the quaternization of non-oxidized amino groups with bromoacetic acid. AZB is licensed and authorized in Russia, the Commonwealth of Independent States, and Slovakia, for infectious and inflammatory diseases^50^. In 2008 the World Health Organization (WHO) assigned the international non-proprietary name (INN) to the drug, Polyoxidonium^®^-AZB^51^. The biological activity is mainly observed in the bridging of inflammatory responses to immune responses, the activation of phagocytic cells, T- and NK-cells, the maturation of DCs, the modulation of the synthesis of cytokines, such as IF-alpha and -gamma, antitoxic activities, and membrane stabilizing effect^50,52^. AZB binding has been shown to occur more rapidly with monocytes and neutrophils than lymphocytes. Additionally, AZB has been shown to enter leukocytes *via* endocytosis and significantly increase the level of intracellular H_2_O_2_ in monocytes and neutrophils, which activates NFkB and the inflammatory and immune response^53^. AZB also has detoxifying and antioxidant properties that are largely determined by the structure and high molecular weight of the drug. AZB has been used as an immune adjuvant in anti-infectious vaccine, particularly in the quadrivalent inactivated subunit adjuvanted influenza vaccine, Grippol^®^ quadrivalent (NPO Petrovax Pharm). Immunization with a single dose of adjuvanted quadrivalent influenza vaccine (QIV) with a decreased amount of hemagglutinin protein to all virus strains due to the use of AZB forms protective immunity in healthy people^54^.

#### Bio-clinical activity in infection

**Figure 5:**
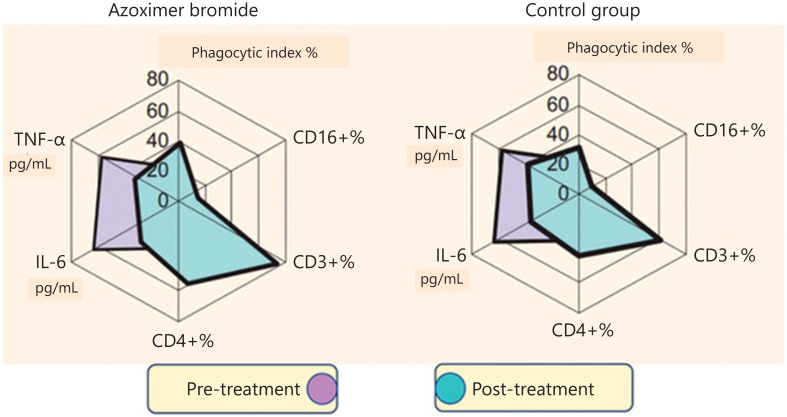
Dynamics of cellular and humoral immunity parameters in patients with mild infection (group I) and severe course (group II) of community-acquired pneumonia before and after treatment, including polyoxidonium. On day 10, circulating CD3+ lymphocytes were significantly increased (*P* < 0.05), rising from 48.47% ± 2.43% to 73.16% ± 2.43% in group I and from 48.70% ± 2.65% to 61.47% ± 3.09% in group II. CD4+ lymphocytes were also significantly increased (*P* < 0.05) from 31.56% ± 1.30 to 45.56% ± 1.59% in group I and from 31.29% ± 1.78% to 41.29% ± 1.82%. CD8+ lymphocytes were significantly increased (*P* < 0.05) from 17.44% ± 0.03% to 25.44% ± 0.03% in group I and from 17.50% ± 1.92% to 20.01% ± 0.92%. No changes were observed for CD19+ lymphocytes^57,58^.

As a medicine, AZB has been widely used, having a good safety profile throughout clinical development and post-marketing surveillance^55^. AZB is administered intramuscularly at 6 mg, twice a week (10 injections), with an oral form as well. Between 1997 and 2017, 439 adults and children with various forms of active tuberculosis were included in 10 clinical studies combining AZB with standard chemotherapy. In a comparative study (25 controls *vs.* 29 treated with AZB), both clinical and radiologic improvement was higher in the group of patients receiving AZB (74% *vs.* 44% and 52% *vs.* 10%, respectively) 1 month after the end of using it (personal data). In non-COVID-19 acute respiratory infections (ARIs), 4 studies have been carried out in adults and children with 2 randomized studies, including 52 with AZB *vs.* 55 controls, suggesting a clinical benefit with a significantly shorter duration of fever (80.13 ± 20.75 *vs.* 100.99 ± 24.91 days, respectively; *P* < 0.001)^56^, and in prophylactic treatment in patients with frequent ARIs with a significant reduction in recurrences of ARIs between patients treated with AZB *vs.* controls (18/90 *vs.* 80/90 patients, respectively)^57^. Immune modulation was observed in 45 patients with community-acquired pneumonia, including 25 with mild infections (group I) and 20 patients with severe infections (group II). As shown in **Figure 5**, a significant increase of CD3+ lymphocytes as well as CD4+ and CD8+ lymphocyte subpopulations were observed in both groups of patients, with a decrease of IL-8 and IL-6 serum levels^57,58^.

AZB (12 mg IV once daily for 3 days then IM every other day until D17) has been prescribed to COVID-19 patients in Slovakia and Russia as part of licensed therapy. Phase II studies involving 84 patients were conducted in Russia and historically compared to the control arm of a randomized study in China with matched bio-clinical characteristics^59,60^. This protocol was reviewed and approved by an Independent Ethical Committee (Pharmnadzor, REC number 229; 9 April 2020). The trial is registered on ClinicalTrials.gov (NCT04542226). In this study, the AZB-treated patient group showed clinical improvement by day 14/15, as measured by the WHO Ordinal Scale (OS) 4.36–2.90 *vs.* OS 3.99–3.87 in the control arm. The mean length of hospitalization was similar in the control group (16.0 days); however, day 28 mortality was higher at 25.0% (*n* = 25). AZB has also been suggested to reduce symptoms of long-COVID-19, particularly chronic fatigue, in a recent comparative cohort study.

#### Clinical activity in cancer

AZB (6 mg every other day for 10 IV injections) plus standard chemotherapy or radiotherapy treatment with usual management has been used in > 500 cancer patients at 2 cancer centers in Russia with bio-clinical data analysis in a real-world study, with historical comparison in addition to standard therapy alone, as shown in **Table 1**.

**Table 1:** Azoximer bromide (AZB) in patients with cancer

Type of cancer	Type of study	*n* patients (pts)	Clinical results		
Melanoma stage I-IV	Cohort study	30 pts with surgery alone 40 pts AZB post-surgery	OS (3 years) 13.3% 92.5%		
Breast stage T1-2 N0 M0	Cohort study	94 pts Adj. Tt to FAC 20 pts neoadjuvant (7D)	Improve tolerance and FACT-G 30% pathomorphologic change, increase of intra-tumoral T-lymphocytes; 1 pt with histologic CR		
NSCLC	Cohort study	194 pts (stage III-IV) 129 CDDP VP16 + AZB 69 pts AZB 4^th^–8^th^ cycles 66 pts AZB 1^st^–8^th^ cycles 65 pts chemotherapy	Late toxicity	Infections	Deaths during CT
			1.77/pt	1.02/pt	*n* = 3
			1.88/pt	0.90/pt	*n* = 2
			3.05/pt	1.39/pt	*n* = 10
28 Children HL LH	Cohort study	16 pts 5 days before CT 18 pts	20%–30% tumor mass reduction		

Different cohort studies were performed in cancer patients (pt), including melanoma after surgery, and compared to a matched population with surgery alone, in non-small cell lung cancer (NSCLC), in children with Hodgkin lymphoma (HL) or Langerhans histiocytosis (LH) and in localized breast cancer with neoadjuvant or adjuvant treatments. In 20 pts having AZB before surgery, comparative histologic aspects were performed including pathomorphologic changes in 30% of the patients with one patient in complete response (CR). CT, chemotherapy; FAC, 5FU, adriamycin, cyclophosphamide; CDDP, cisplatin; VP16, etoposide; pt, patient; Tt, treatment; OS, overall survival; FACT-G, Functional Assessment of Cancer Therapy-General, Quality of life general questionnaire.

A reduction in the rate of infection related to chemotherapy or surgery has been also observed in different cancers, including lung cancers, melanoma, breast cancer, and colon cancer. One hundred ninety-four patients (mean age, 53.7 ± 3.9 years; range, 30–73 years) with advanced lung cancer (stage III-IV), mainly squamous cell type (64.9%) and adenocarcinoma (25.3%), were treated at the Bashkir State Medical University in Ufa (Bashkortostan, Russia)^61^. All patients received cisplatin (60 mg/m^2^ IV on day 1) and etoposide (120 mg/m^2^ IV on D1 and D3). Peripheral lung cancer was found in 70.6% of patients and central lung cancer was diagnosed in 29.4% of patients. One-hundred twenty-nine patients received AZB (6 mg IM for 5 courses) with concomitant chemotherapy, one-half of the patients receiving AZB from the 1st–8th cycles (subgroup 1) and the other half from the 4th–8th cycles of chemotherapy (subgroup 2). The first two AZB injections were administered before the start of the chemotherapy cycle, and the following 3 injections were administered during the CT cycle. The control group of 65 patients received only chemotherapy. In both groups of patients who received AZB, late toxic complications were reduced (3.05 per patient in the control group *vs.* 1.88 per patient in subgroup 1 and 1.77 in subgroup 2). Patients with AZB had a significantly lower incidence of infection, especially for severe infections (*P* < 005). Additionally, the mortality rate was also significantly reduced in the 2 subgroups receiving AZB, with 10 patients dying in the comparison group *vs.* 2 and 3 in subgroups 1 and 2, respectively. The full dose of chemotherapy was administered to 75.8% of patients in subgroup 1 and 63.5% in subgroup 2 *vs.* 58.5% in the control group. Thus, AZB reduced the complications of chemotherapy, particularly infections, but also a reduction in metastasis in localized lung cancer has been suggested when patients received AZB after surgery^53^. A survival benefit was also suggested in a retrospective analysis on the 3-year survival of 70 patients with grade l-IV cutaneous melanoma and 13.3% in the group having undergone surgery alone and 92.5% in the group of patients receiving AZB after surgery^62^.

Extensive documentation has been done on immune effects *in vivo*, with a significant increase in circulating CD3, CD4, CD8 T- and B- and CD45RA+ cells^61^. In 21 patients with chronic lymphocytic leukemia, increased T-cell counts, neutrophil phagocytic activity, and immunoglobulin levels were also observed^50^. Changes in the composition of infiltrating lymphocytes have been observed in breast cancer after neoadjuvant treatment, with complete eradication of tumor cells in a patient with triple negative breast cancer^50,52^. Recently, 243 patients with soft tissue sarcoma (STS) and 1391 patients with melanoma were retrospective analyzed at Petrov National Medical Research Centre of Oncology (St. Petersburg, Russia) Among the 25 patients with STS and 42 patients with melanoma who received AZB, treatment with AZB emerged as a favorable prognostic factor using a Cox proportional-hazards model for TTP analysis (HR = 0.475 and 0.547, respectively)^63,64^. Additionally, combined therapy (AZB plus DC vaccine pulsed with autologous tumor) showed good tolerance in 50 patients with advanced disease, including 32 patients with melanoma and 18 patients with STS.

## Conclusions

More than immune adjuvants, there is a need for immune modulators in cancer that are particularly active in modifying the TME to break the TME down and to reverse pro-cancer activity. Among these functions, the link between innate and adaptive responses constitutes the main process, including the process of antigen presentation. In the era of immune checkpoint inhibitors, CAR T-cells, bi-specific monoclonal antibodies, and anticancer vaccine, there is a need for therapeutic combinations, and the rediscovery of certain unknown or forgotten immune modulators is necessary.
